# Genetic association studies in critically ill patients: a systematic review

**DOI:** 10.1016/j.ebiom.2025.105678

**Published:** 2025-03-31

**Authors:** Wenbo Zhang, Nam Nguyen-Hoang, Sean C.S. Rivrud, Anne-Fleur Zandbergen, Yalan Yan, Eline G.M. Cox, Eva Suarez-Pajes, Amanda Y. Chong, Alexander J. Mentzer, Carlos Flores, Gerton Lunter, Frederik Keus, Harold Snieder

**Affiliations:** a Department of Epidemiology, University of Groningen, University Medical Center Groningen, 9713 GZ, Groningen, the Netherlands; b Department of Critical Care, University of Groningen, University Medical Center Groningen, 9713 GZ, Groningen, the Netherlands; c Department of Clinical Pharmacy, University of Michigan College of Pharmacy, Ann Arbor, MI, USA; d Department of Critical Care Medicine, The Second Affiliated Hospital of the Chinese University of Hong Kong (Shenzhen) (Longgang District People's Hospital of Shenzhen), Shenzhen, 5l8100, China; e Research Unit, Hospital Universitario Nuestra Señora de Candelaria, Instituto de Investigación Sanitaria de Canarias (IISC), 38010, Santa Cruz de Tenerife, Spain; f The Centre for Human Genetics, Nuffield Department of Medicine, University of Oxford, Oxford, UK; g Ludwig Institute for Cancer Research, Nuffield Department of Medicine, University of Oxford, Oxford, UK; h Big Data Institute, Li Ka Shing Centre for Health Information and Discovery, University of Oxford, Oxford, UK; i CIBER de Enfermedades Respiratorias (CIBERES), Instituto de Salud Carlos III, 28029, Madrid, Spain; j Genomics Division, Instituto Tecnológico y de Energías Renovables (ITER), 38600, Santa Cruz de Tenerife, Spain; k Faculty of Health Sciences, University of Fernando Pessoa Canarias, 35450, Las Palmas de Gran Canaria, Spain

**Keywords:** Critically ill patients, Intensive care, Genetics, Genome-wide association study, Candidate gene study

## Abstract

**Background:**

Critical illness is complex, and genetic research holds the potential to uncover underlying disease mechanisms. However, existing research results have not been systematically summarized. This study aims to compile all genetic association studies in critically ill patients and assess their risks of bias.

**Methods:**

We systematically reviewed PubMed, EMBASE, and the Cochrane Library (PROSPERO protocol: CRD42021209744) and conducted a bias risk assessment of identified studies using a newly developed risk-of-bias assessment tool for genetic association studies. We compiled all significant single nucleotide polymorphisms (SNPs) from the studies identified in our systematic review and conducted a lookup in the results of two genome-wide association studies (GWASs) in critically ill patients.

**Findings:**

We identified a total of 61 studies that evaluated genetic variants with various traits in adult intensive care patients, encompassing a total of 126 genetic loci and focussing on six clustered critical care-related traits. Assessment of the risk of bias across these studies revealed that 97% of the studies demonstrated some concerns or high risk of bias and only two studies (3%, both GWAS), had an overall low risk of bias. Only two significantly associated SNPs emerged from the two studies assessed to have a low risk of bias. One SNP (rs4957796) in *FER* was associated with 28-day survival (*p* = 3.4 × 10^−9^) and mortality (*p* = 5.6 × 10^−8^), and rs9508032 in *FLT1* was associated with respiratory failure (*p* = 5.2 × 10^−8^). Fifty-four significant associations were replicated in the candidate gene studies, but most had an overall high risk of bias. Only one association, of rs2569190 in *CD14* with mortality, remained significant after multiple testing correction.

**Interpretation:**

Our systematic review underscored a deficiency in high-quality genetic research in critical care medicine. The detected gaps emphasize an urgent need for additional rigorous genetic association studies that are preferably genome-wide, to enhance our understanding of the underlying mechanisms of critical illness.

**Funding:**

Wenbo Zhang was financially supported by a grant from the 10.13039/501100004543China Scholarship Council (File no. 202006210041). Carlos Flores was funded by 10.13039/501100004587Instituto de Salud Carlos III (PI23/00980 and CB06/06/1088) and co-financed by the 10.13039/501100008530European Regional Development Fund, “A way of making Europe” from the European Union; and the agreement OA23/043 with Instituto Tecnológico y de Energías Renovables (ITER) to strengthen scientific and technological education, training, research, development and scientific innovation in genomics, epidemiological surveillance based on massive sequencing, personalized medicine, and biotechnology. Eva Suarez-Pajes was financially supported by Agencia Canaria de Investigación, Innovación y Sociedad de la Información de la Consejería de Economía, Conocimiento y Empleo y por el 10.13039/501100004895Fondo Social Europeo (FSE) Programa Operativo Integrado de Canarias 2014–2020, Eje 3 Tema Prioritario 74 (85%) (TESIS2022010042).

**Research in context**

**Evidence before this study**

Previous studies have explored the associations between genetic variants and clinical outcomes in specific subgroups or syndromes of critically ill patients, but these findings have not been systematically reviewed or synthesized. Additionally, the quality of these studies varies widely, and there has been no thorough assessment of their risk of bias.

**Added value of this study**

Our study systematically reviewed and evaluated the quality of all genetic association studies in critically ill patients irrespective of the admitting diagnoses or syndromes for classifying organ dysfunction. We identified 61 studies examining the relationships between genetic variants and various critical care-related traits across 126 genetic loci. Our rigorous risk-of-bias assessment revealed that 97% of these studies had some concerns or a high risk of bias, with only two genome-wide association studies showing a low risk of bias. We found significant associations between certain genetic loci and critical outcomes, such as rs4957796 in *FER* and rs2569190 in *CD14* associated with mortality, and rs9508032 in *FLT1* with respiratory failure.

**Implications of all the available evidence**

This study highlights both the potential and the challenges of genetic research in critical care medicine. While some significant associations were identified, most studies have a high risk of bias and lack replication. This underscores the urgent need for rigorously well-designed genetic association studies. Genome-wide approaches are especially needed to better understand the mechanisms underlying critical illness.

## Introduction

Critically ill patients in the intensive care unit (ICU) are characterized by considerable heterogeneity in terms of the diagnosis upon admission and course of disease. The extent and severity of organ failure and outcomes may be diverse,^1^ even when sharing a common initial diagnosis. This variability underscores the multifaceted trajectories that critical illness may traverse, ranging from rapid recovery to progression toward multi-organ failure and death. Further, “unrelated” acute critical illnesses may exhibit remarkable similarities in disease progression and patterns of organ failure, irrespective of the initial primary disease.^2^ Such complexities pose substantial challenges for interventions in critically ill patients.

Over the past two decades, genetic research has emerged as a promising approach in medicine. Various disciplines have integrated genetic studies alongside traditional clinical studies, seeking to illuminate an understanding of underlying disease mechanisms.^3^ Concurrently, researchers have successfully identified common genetic variants associated with a wide range of diseases, offering insights into the underlying mechanisms of complex diseases. Moreover, in select medical fields, researchers have begun combining genetic data with individual patient profiles, facilitating the implementation of individually tailored interventions to improve patient outcomes, as observed in fields such as oncology, cardiology, neurology, and pharmacogenomics.^4^, ^5^, ^6^, ^7^

In addition to these areas, the field of critical care medicine has also seen a growing interest in the application of genetic research. Despite these advancements, the large majority of genetic association studies have focused on assessing risk factors within specific diseases, syndromes or outcomes within critical care, such as sepsis, acute respiratory distress syndrome (ARDS), or acute kidney injury.^8^, ^9^, ^10^, ^11^ These studies failed to identify common genetic variants associated with different disease outcomes. Moreover, certain studies have not strictly confined their inclusion criteria to critically ill patients. Typically, these studies included control groups comprising the general population, potentially failing to accurately capture issues specific to critical care medicine.^12^ Overall, there exists a gap in comprehensive evaluations encompassing all available genetic evidence across a spectrum of diseases and outcomes prevalent in critical care medicine, including (multiple) organ failure, sepsis, and mortality.

From a cursory reading of the literature,^10^^,^^12^ it appears that the predominant body of genetic evidence in critical care medicine comprises candidate gene studies. As is well known, such studies often harbour design flaws that may introduce biases.^13^^,^^14^ However, the full body of research may still contain independently replicated and trustworthy findings. Therefore, we conducted a systematic review to summarize all existing genetic association studies in critically ill patients and assess the risk of bias associated with each study.

## Methods

### Ethics

Ethical approval and consent to participate were not applicable as this was a systematic review of existing studies. All included studies had reported approvals from ethical committees.

### Data source and search strategy

This systematic review was conducted according to the pre-published protocol^15^ and the *HuGE Review Handbook V1.0* (guidelines for systematic review and meta-analysis of gene disease association studies)^16^^,^^17^ and reported following the Preferred Reporting Items for Systematic Reviews and Meta-analyses (PRISMA) (Supplementary File 1, Table S1).^18^ This study is registered with PROSPERO number: RD42021209744.

A comprehensive search was conducted across multiple databases, including PubMed, EMBASE, and the Cochrane Library, utilizing standardized vocabulary and free text to ascertain studies involving critically ill patients afflicted with a spectrum of diseases and organ failures, including but not limited to respiratory failure, acute kidney injury, sepsis and multiple organ failure. The latest search was performed on December 18, 2024. Detailed search strategies for each database are delineated in Supplementary File 1, Table S2.

### Inclusion and exclusion criteria

All types of genetic association studies, encompassing genome-wide association studies (GWAS) and candidate gene studies, were considered for inclusion in this systematic review. Exclusion criteria were studies lacking original data (e.g., review studies), conference abstracts, case reports/series, editorials, and studies not conducted on human subjects. Furthermore, studies focussing on (genome-wide) methylation or gene expression were also excluded.

Eligible studies were restricted to those involving adult patients (≥18 years old) who were critically ill and admitted to an ICU. Specifically, studies addressing the development of various diseases, syndromes or outcomes were included, such as (multiple) organ failure, heart failure, liver failure, acute kidney injury, renal insufficiency, sepsis, septic shock, ARDS, pulmonary oedema, acute lung injury, and COVID-19 as long as the large majority of the patients included in the studies were admitted to an ICU.^15^ Conversely, studies involving patients with conditions other than those specified (e.g., neurological diseases like stroke or subarachnoid haemorrhage) were excluded, as the majority of these patients typically do not require an ICU admission. Furthermore, cohorts of patients admitted to the ICU following planned surgeries were excluded, while those involving patients admitted to an ICU after acute surgery were considered for inclusion. (Supplementary File 1, Table S3).

Throughout the screening process, and in contrast to our expectations, we found that a significant portion of studies exhibited exceedingly small sample sizes. Consequently, an additional exclusion criterion was introduced, stipulating a minimum total sample size (including discovery and replication samples) of 300 patients. Similar criteria have been used previously (sample size <400).^12^

### Identifying studies

All publications identified across the three databases were imported into Endnote, with duplicate entries eliminated by the software. Subsequently, a team of six reviewers (WZ, NNH, SR, AZ, YY, EC) scrutinized the titles and abstracts of each study to ascertain eligibility in accordance with the pre-specified selection criteria. Upon preliminary selection, five reviewers independently assessed the full-text articles of the identified publications, with each article being assessed by two reviewers to ensure alignment with all inclusion criteria. Any discrepancies arising during this process were discussed and resolved, with recourse to three senior reviewers (GL, FK, HS) in cases of disagreement. Ultimately, a database was created for the systematic data extraction of eligible studies in duplicate, independently. The PRISMA flow chart was used to document the number of hits screened, full-text studies retrieved, and excluded studies.^18^

### Risk of bias assessment

For evaluating the risk of bias of individual studies, we employed a quality assessment tool derived from the *HuGE Review Handbook V1.0* and its updated version integrating content specific to GWAS.^16^^,^^17^ This tool encompasses four domains: “selection bias”, “information bias”, “confounding”, and “multiple testing and replication”, and aims to comprehensively evaluate the risk of bias in each study. Details of the quality assessment tool are shown in Supplementary File 1, Table S4 and were published in the protocol paper.^15^ The assessment process involved six reviewers (WZ, NNH, SR, AZ, YY, EC) evaluating the risk of bias in all included studies, in duplicate and independently. In cases of discrepancies or uncertainties, discussions were held among the reviewers to reach a consensus and resolve any disagreements, ensuring the integrity and accuracy of the assessment.

### Data extraction

A standardized data collection form facilitated the extraction of key information from full-text reports, encompassing study characteristics such as title, first author, publication year, journal, country of study, study design, and risk of bias. Population characteristics, including the number of patients, age distribution, male ratio, ethnicity, and disease severity scores, were also captured. Main outcomes and details regarding (candidate) genes and genetic markers, including genotyping methods, type of genetic markers, effect sizes, and *p*-values, were meticulously recorded. However, due to the prevalent lack of comprehensive information in many studies, particularly regarding simultaneous hypothesis testing, we did not conduct adjustments of *p*-values for studies that did not account for multiple comparisons. Instead, *p*-values reported in the studies were utilized directly.

To ensure consistency in the extraction of *p*-values, a standardized approach was adopted. In cases where studies simultaneously reported both discovery and replication results without amalgamating them, the *p*-values from the discovery stage were adopted as the final values. Conversely, studies identified as “replication studies” that solely provided results from the replication stage were treated accordingly, with replication stage *p*-values considered final. Furthermore, in instances where studies conducted meta-analyses on both discovery and replication results to derive joint effect sizes and *p*-values, the meta-analysis-derived *p*-values were deemed final.

If an original study only mentioned the single nucleotide polymorphism (SNP) but not the gene, we identified the corresponding gene through the SNPdb database.^19^

### Statistics

A comprehensive description of all studies was conducted and the key findings of each study were reported. This description included an overview of the study designs employed and the characteristics of the patient cohorts under investigation. Additionally, we documented the genetic loci and genetic markers identified as being associated with specific traits, categorizing them based on the study design utilized (candidate gene study without replication, candidate gene study with replication, or GWAS). Due to the diversity in the exact outcome definitions across studies, we clustered these into six critical care-related outcome categories: mortality/survival, respiratory failure, infection/sepsis, kidney failure, multiple organ failure, and other traits. The specific phenotypes included in each category can be found in Supplementary File 2. Table S1 provides a summary of the key characteristics of all the included studies. The Kruskal–Wallis test was used to evaluate whether there were statistical differences in the sample size, male proportion, and median age of studies across different study designs. More detailed information on each study is available in the Supplementary Files 3 and 4.

Finally, to test whether the significant associations reported in the candidate gene studies (*p* < 0.05) and/or GWASs (*p* < 1 × 10^−7^) identified in our systematic review could be replicated in (other) GWASs, we compiled a list of SNPs for which at least one significant association was reported within any of the above critical care-related outcome categories and conducted lookups in the summary statistics of the two GWASs included in the review. One of the GWASs focused on 28-day survival in patients with sepsis due to pneumonia^20^ and the other on sepsis-associated ARDS.^21^ For SNPs reported in GWAS, we performed a cross-check by querying SNPs from one GWAS in the summary statistics of the other. Lookup results that showed *p* < 0.05 and consistent effect direction were considered replicated with nominal significance. We also corrected for multiple testing by applying a Bonferroni correction (*p* < 0.05 divided by the total number of significant SNPs queried). All analyses were conducted in R 4.0.5.

### Deviations from protocol

In our protocol, we initially did not impose any restrictions on the sample size of the studies. However, as we conducted our screening process, we encountered a substantial number of studies with exceedingly small sample sizes, realizing that these results would by definition be considered unreliable. Therefore, in an effort to ensure the reliability of the outcomes, we introduced an additional exclusion criterion: studies with a sample size less than 300 (encompassing both the discovery and replication cohorts) were excluded during the screening process.

### Role of funders

The funders had no role in the study design, data collection, analysis, interpretation of data, decision to publish, or preparation of the manuscript.

## Results

### Search results

**Fig. 1:**
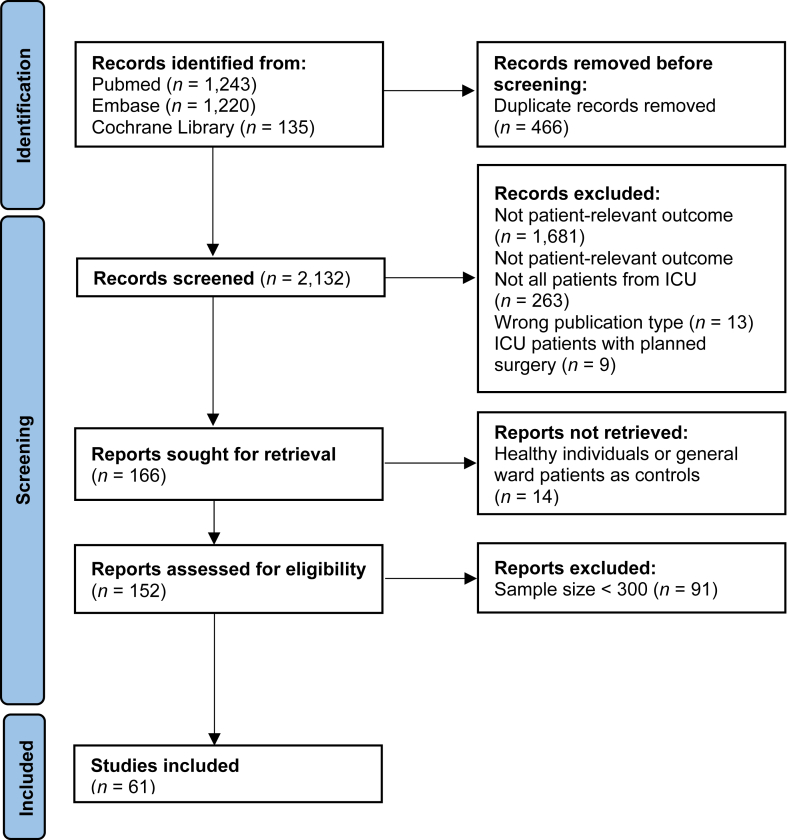
Systematic review flowchart.

The search strategy resulted in the identification of a total of 2598 studies, as illustrated in Fig. 1. Following the removal of 466 duplicates and the exclusion of 1966 studies due to the screening of titles and abstracts based on exclusion criteria, we proceeded to retrieve and assess the full texts of 166 studies against the comprehensive selection criteria. Subsequently, an additional 14 studies were deemed ineligible for inclusion based on full text. Further, after applying our newly introduced sample size criterion (*n* > 300 patients), an additional 91 studies were excluded from further consideration. Detailed information regarding these 14 and 91 studies can be found in Supplementary File 5. Ultimately, a total of 61 studies met all inclusion criteria and underwent comprehensive analysis as part of this study.

### Characteristics of the studies

In Table 1, there were 2 GWASs, 19 candidate gene studies with replication, and 40 candidate gene studies without replication among these 61 studies. The median sample size was 733 with an interquartile range (IQR) of 529–1451. The median sample sizes for the three kinds of studies with different designs (GWAS, candidate gene studies with and without replication) were 2736, 887, and 651, respectively (Kruskal–Wallis test: *p* < 0.01). The proportion of males in these studies was larger than that of females with a median of 63.8% (IQR: 60.3%–71.3%). The sex distributions were similar across different study designs as well (Kruskal–Wallis test: *p* = 0.87). The IQR of participants’ ages was from 45 to 63 years, with a median of 59 years. The results indicated that there was no significant difference in age distribution among different study designs (Kruskal–Wallis test: *p* = 0.58).

**Table 1:** Characteristics of the included studies.

	Overall *n* = 61	GWAS *n* = 2	Candidate gene study with replication *n* = 19	Candidate gene study without replication *n* = 40	*p*
Sample size, *n* (IQR)	733 (529–1451)	2736 (2335–3136)	887 (693–1632)	651 (500–892)	<0.01
Male proportion (IQR)	63.8% (60.3–71.3)	61.9% (59.7–64.1)	63.6% (61.9–68.0)	64.0% (60.3–75.4)	0.87
Median age of studies, *yrs* (IQR)	59 (45–63)	63 (63–63)	57 (44–63)	60 (46–63)	0.58
Reported associations, *n* (number of loci)	306 (126)	5 (5)	167 (77)	134 (55)	
Mortality/survival	101 (69)	1 (1)	57 (45)	43 (28)	
Respiratory failure	91 (40)	4 (4)	61 (29)	26 (13)	
Infection/sepsis	45 (28)	0	13 (6)	32 (22)	
Kidney failure	41 (30)	0	28 (23)	13 (8)	
Multiple organ failure	24 (21)	0	7 (6)	17 (15)	
Other traits^a^	4 (4)	0	1 (1)	3 (3)	

IQR, Interquartile range.

a Other traits include “body temperature”, “delirium during sepsis” and “length of hospital stay after ICU”, and “length of ICU stay”.

**Fig. 2:**
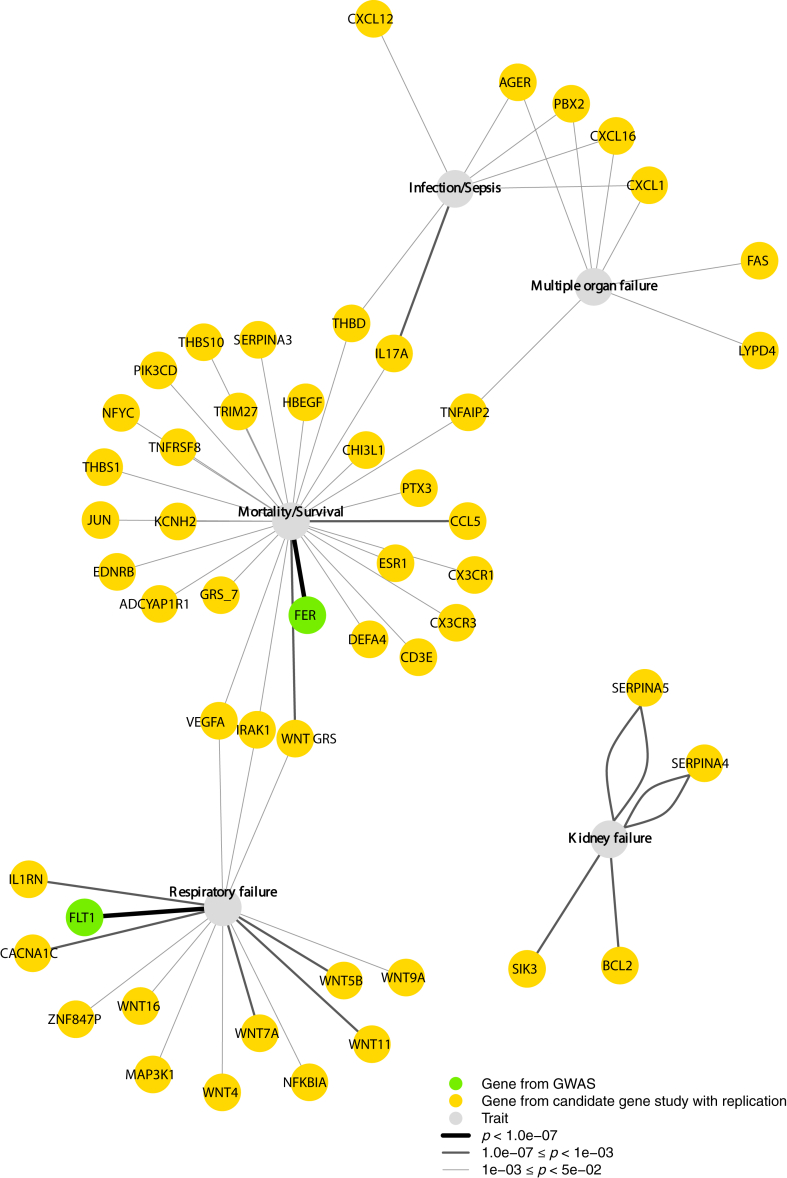
Significant associations between genetic loci and clustered traits in critical care medicine. GRS, Genetic risk score. The “GRS_*7genes*_” contained seven genes, including the genes *CCL5*, *CX3CR1*, *DEFA4*, *ESR1*, *NFYC*, *SERPINA,* and *TNFRSF8*. The “GRS_*WNT*_” contained eight single nucleotide polymorphisms related to *WNT* gene family, including the genes *WNT4*, *WNT5B*, *WNT7A*, *WNT9A*, *WNT11*, and *WNT*. “Other traits” in the figure includes “body temperature”, “delirium during sepsis” and “hospital stay after ICUs”. Each line represents one study included. The figure contains some “*Gene1*-*Gene2*” dots, which means this locus is close to both adjacent genes.

The 61 studies reported a total of 306 associations of genetic loci with outcome traits, involving 126 unique loci. These associations encompassed various traits, with 101 associations related to mortality/survival, involving 69 loci; 91 associations related to respiratory failure, involving 40 loci; 45 associations related to infection/sepsis, involving 28 loci; 41 associations related to kidney failure, involving 30 loci; 24 associations related to multiple organ failure, involving 21 loci; and 4 associations related to other traits, including body temperature, delirium during sepsis, length of hospital stay after ICUs, and length of ICU stay, involving 4 loci. Robust results reported by GWAS or replicated candidate gene studies were shown in Fig. 2. Supplementary File 6 shows all the significant results.

### Risk of bias

**Fig. 3:**
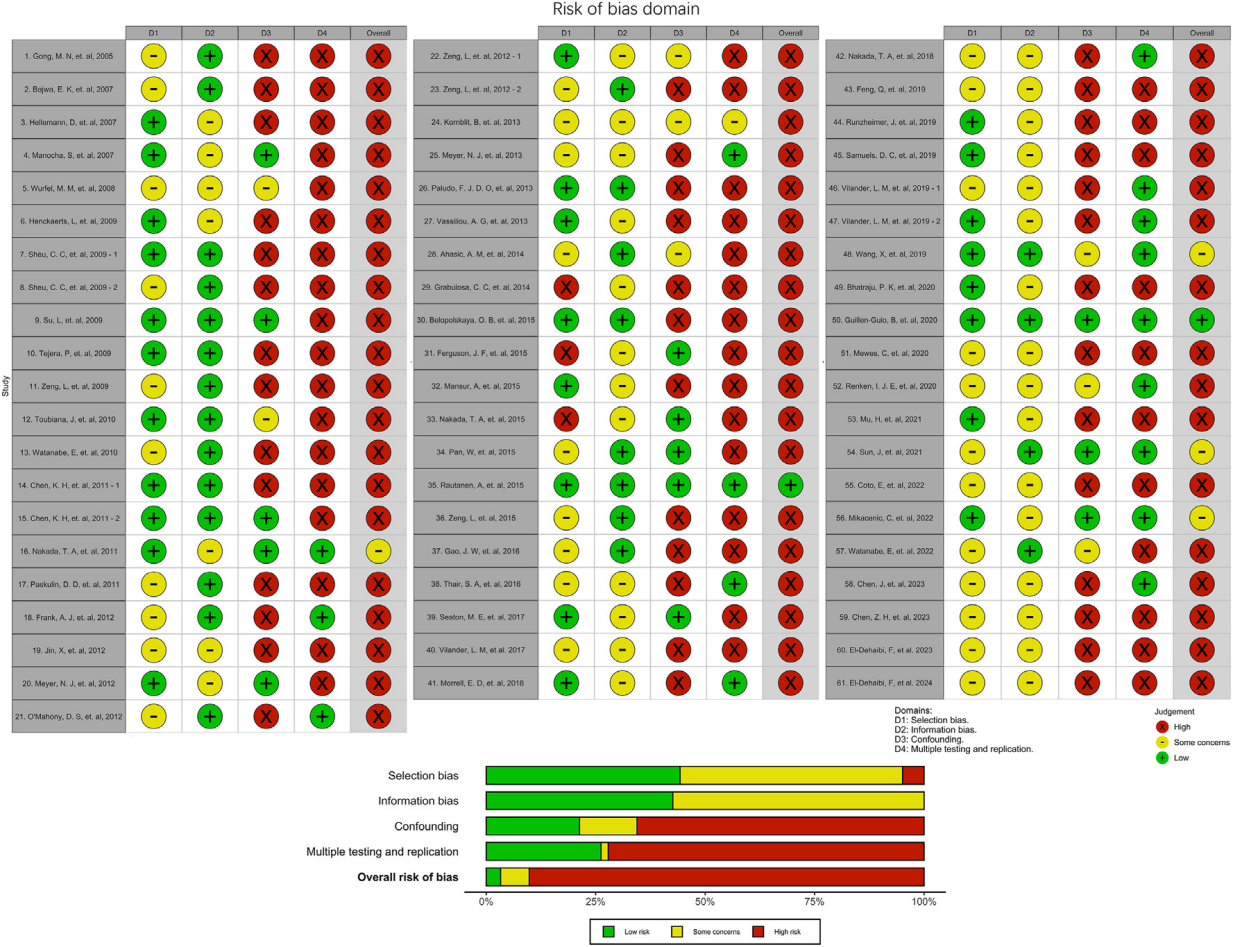
Risk of bias assessment.

The overall risk of bias was high, with 90% of all the studies (55 of 61) assessed as having an overall high risk of bias, 7% of the studies were assessed as having some concerns of risk of bias (4 of 61), and only 3% of all studies were assessed as having overall low risk of bias (2 of 61) (Fig. 3). The most frequent sources of risk of bias were identified in the domain of “selection bias” in 5% (3 of 61) of studies, the domain “confounding” in 66% (40 of 61), and the domain “multiple testing and replication” in 72% (44 of 61). The two studies with a low risk of bias were both GWASs (Fig. 3).

### Significant associations between different loci and traits in critical care medicine

The significant associations between different genetic loci and critical care traits are described in Fig. 2. Among all the 126 genetic loci, 91 loci (72%) were significantly associated with one or more specific outcomes. In these 91 loci, 49 loci (54%) were reported to be associated with mortality/survival, 28 loci (31%) with respiratory failure, 23 loci (25%) with infection/sepsis, 18 loci (20%) with multiple organ failure, 9 loci (10%) with kidney failure, and 3 loci (3%) with other traits. Notably, 43 loci (47%) were linked to more than one clustered trait simultaneously. For example, *CD14* was associated with mortality/survival and multiple organ failure; *HMOX1* was associated with kidney failure and respiratory failure; *TNF* was associated with mortality/survival and respiratory failure.

Of the 91 significant loci, two were identified through GWAS involving two SNPs. One SNP (rs4957796) in *FER* was associated with both 28-day survival (Cox regression: *p* = 3.4 × 10^−9^) and mortality (logistic regression: *p* = 5.6 × 10^−8^),^20^ and the other SNP (rs9508032) in *FLT1* was associated with respiratory failure (logistic regression: *p* = 5.2 × 10^−8^).^21^

Sixty-nine significant associations between genetic loci and the clustered traits originated from candidate gene studies with replication, including associations such as ADCYAP1R1 with survival,^22^ *BCL2* with kidney failure,^23^ and *CXCL1* with infection/sepsis.^24^ Although these results were significant and involved replication, the overall risk of bias in these associations was mostly high (59 of 69) or of some concerns (10 of 69). Details can be found in Supplementary File 3.

### Candidate gene study results in GWAS summary statistics

We included two GWASs in our systematic review. Rautanen et al. conducted a GWAS on 28-day survival in patients with sepsis due to pneumonia, identifying one genome-wide significant SNP,^20^ and Guillen-Giuo et al. performed a GWAS on sepsis-associated ARDS in patients, reporting one significant SNP^21^ as mentioned above. In total, 59 candidate gene studies investigated 165 SNPs and 88 SNPs showed at least one significant result with an outcome trait (Supplementary File 3). Among these 88 SNPs, rs2569190 (*CD14*, Cox regression: *p* = 3.9 × 10^−4^) and rs1800972 (*DEFB1*, Cox regression: *p* = 4.3 × 10^−2^) were significantly associated with 28-day survival in patients with sepsis due to pneumonia in the GWAS and rs1800629 (*TNF*, logistic regression: *p* = 2.0 × 10^−2^) was significantly associated with sepsis-associated ARDS with all three SNPs having the same effect direction as in the original candidate gene studies (Table 2). Cross-checking the GWAS SNPs yielded no significant results (Supplementary File 1, Table S4).

**Table 2:** GWAS summary statistics for significant SNPs in the candidate gene studies and validated in two GWASs.

rsID	Gene	Trait	Chromosome	Position	Effect allele	Non effect allele	Effect allele frequency	Effect size	Standard error	*p*^a^
rs2569190	*CD14*	Survival from sepsis due to pneumonia	5	140,012,916	A	G	0.49	0.80	0.23	3.9 × 10^−4^
rs1800972	*DEFB1*	Survival from sepsis due to pneumonia	8	6,735,423	C	G	0.20	0.37	0.18	4.3 × 10^−2^
rs1800629	*TNF*	Sepsis-associated ARDS	6	31,543,031	A	G	0.15	−0.41	0.18	2.0 × 10^−2^

ARDS, Acute respiratory distress syndrome.

The information of each SNP is based on the *GRCh37* reference genome.

a The Bonferroni-corrected *p*-value threshold was 5.8 × 10^−4^.

After applying the Bonferroni correction (*p* < 0.05/88), only rs2569190 in the *CD14* gene remained significant. In the original candidate gene study, rs2569190 was significantly associated with 30-day survival in sepsis patients (Cox regression: *p* = 2.8 × 10^−2^).^25^

## Discussion

We identified 61 studies evaluating genetic variants associated with various traits in critically ill patients, covering 126 genetic loci and focussing on six clustered critical care-related traits. Our risk of bias assessment revealed that 97% of the studies had some concerns or high risk of bias, with only two GWAS studies (3%) showing low risk. Two SNPs emerged from the GWASs: rs4957796 in *FER* was associated with survival (Cox regression: *p* = 3.4 × 10^−9^), and rs9508032 in *FLT1* was associated with respiratory failure (logistic regression: *p* = 5.2 × 10^−8^). While 69 significant associations were found in candidate gene studies with replication, most had high risk of bias. Only three significant associations were validated in GWAS lookups, with rs2569190 in *CD14* remaining significant for 28-day survival in sepsis patients after multiple testing correction.

From the perspective of gene–disease associations, our comprehensive systematic review covered genetic studies in critical care medicine. Nakada et al. explored whether similar cardiovascular disease and sepsis risk genes share similar drug targets in a review.^12^ The researchers listed 24 previously confirmed genes associated with sepsis and cardiovascular diseases. Some findings are consistent with our study, for instance, *IL17A, IL1B, MBL, NOD2, PPARG* and *SOD2* genes were associated with sepsis. *IL17A, IL1B, MBL,* and *NOD2* were also reported to be associated with mortality, and *PPARG* was associated with multiple organ failure in our study (Supplementary File 3). However, this review did not limit the study population to critically ill patients as ours did, which may explain why we did not find these genes to be associated with both sepsis and cardiovascular diseases. Additionally, they did not assess the risk of bias in each included study.

In this systematic review, we found that the *FER* gene (rs4957796) was significantly associated with 28-day survival in patients with sepsis due to pneumonia and the *FLT1* gene (rs9508032) with sepsis-associated ARDS. The *FER* gene encodes a non-receptor tyrosine kinase that functions downstream of cell-surface receptors for growth factors.^26^ Additionally, the *FER* gene influences leucocyte recruitment and intestinal barrier dysfunction in response to bacterial lipopolysaccharide,^27^^,^^28^ which may explain its association with sepsis survival. Rogne et al. in a population-based study of 69,294 individuals with 23 years of follow up found the CC genotype of rs4957796 in *FER* to be significantly associated with an increased risk of contracting a bloodstream infection, but—at the same time—with a reduced risk of dying from one,^29^ which is in line with the GWAS finding in Rautanen et al.^20^ Hinz et al.^30^ also replicated Rautanen et al.'s GWAS finding^20^ in a study on Caucasian ICU patients, showing a significant association between *FER* (rs4957796, Cox regression: *p* = 5 × 10^−3^) and 90-day survival. Although their study was excluded from our review due to its small sample size (*n* = 274), it supports the link between *FER* and mortality in critically ill patients. The *FLT1* gene encodes the vascular endothelial growth factor (VEGF) receptor 1, a tyrosine-protein kinase that binds to VEGF-A, and placental growth factor. Although VEGF was initially characterized as a vascular permeability factor,^31^ it also plays a crucial role in the fibroproliferative phase of ARDS^32^ and the resolution of ventilator-associated pneumonia.^33^ A large multi-ancestry GWAS by Shrine et al. found a significant association in the same gene (*FLT1*) with lung function (mixed models in BOLT-LMM or SAIGE: *p* = 2 × 10^−9^).^34^ However, the reported SNP rs11617354 is in very low linkage disequilibrium with rs9508032 (*r*^2^ = 0.005),^35^ indicating an independent signal. Since lung function is related to respiratory failure, this finding nonetheless supports the potential relevance of variation in this gene for critically ill patients.

In our GWAS lookups, only rs2569190 in the *CD14* gene was successfully validated. This gene encodes a crucial co-receptor, which plays a key role in activating monocytes in response to lipopolysaccharides and gram-positive cell wall components like peptidoglycan.^36^ The *CD14* gene has been proved to be associated with mortality.^25^^,^^37^ Extensive research also demonstrates *CD14*'s pivotal role in initiating and maintaining the pro-inflammatory response during sepsis,^38^^,^^39^ which is essential for combating infections and preventing secondary infections in sepsis patients.^40^ Sepsis is a serious condition with a high mortality rate in critically ill patients.^41^ In studies excluded due to small sample sizes, we found that Luiz et al. (*n* = 85) reported a significant association between rs2569190 and survival in critically ill patients.^42^ Although their sample size was small, it can still be considered a successful replication.

Regarding disease prediction, most prognostic models of critical care lack genetic predictive factors.^43^ Presently, commonly used prognostic models in critical care medicine, such as SOFA, SAPS II, APACHE IV,^44^, ^45^, ^46^ predominantly rely on traditional clinical variables. These predictive variables are typically composed of information collected from electronic medical record systems or routine laboratory tests. Their advantage lies in the ease of collection and rapid prediction of patient conditions. However, these models have a significant limitation: they do not incorporate genetic information, which could enhance their predictive accuracy and provide a more personalized approach to patient care. It is hoped that future studies will integrate genetic variables into predictive models offering new insights into critical care medicine.

Regarding COVID-related studies, we did identify a number of them during our search. Only one study^47^ met our inclusion criteria, but it did not report any significant results. The other studies either included patients with mild COVID-19 (non-ICU patients) or had sample sizes smaller than 300. Further details can be found in Supplementary Files 3 and 5.

Our study has summarized the existing genetic evidence within critical care medicine. Moving forward, researchers seeking to delve into the underlying mechanisms of critical illness may find valuable insights in our systematic review. We also hope that the significant findings in our study can be further validated in other independent cohorts. Moreover, our findings underscore a predominant reliance on candidate gene studies fraught with lack of replication and high risk of bias. This highlights an urgent need for additional rigorous genetic association studies that are preferably genome-wide.^48^, ^49^, ^50^ Lastly, our finding that 43 genetic loci (47% of all observed statistically significant loci) were associated with more than one clustered trait simultaneously suggests that there are common mechanistic pathways leading to different modes and severities of organ failure.

We conducted a comprehensive systematic review of genetic studies in critically ill patients. Our stringent criteria for inclusion and exclusion ensured the incorporation of studies involving critically ill patients, thus mitigating any influence from healthy control groups or other hospitalized patients on the results. Building upon the *HuGE Review Handbook V1.0*^16^ and its updated version,^17^ we developed a generally applicable quality assessment tool to evaluate the risk of bias of genetic studies, enabling a risk of bias assessment reflecting the true quality according to current standards of genetic association studies.^15^

We acknowledge several limitations. First, considering the heterogeneity in patient inclusion criteria and disease definitions across various studies, we were unable to conduct meta-analyses for identified associations. Although currently infeasible due to lack of suitable genetic data in the ICU literature, future meta-analytical studies could address this heterogeneity by categorizing ICU patients by admission causes and performing subgroup analyses or meta-regressions for more reliable disease-specific evidence. Second, the presence of publication bias may have led to the omission of unpublished studies with nonsignificant results, potentially inflating the number of significant results and their reported effect sizes. Third, the quality assessment tool has not been applied previously and although it aims to comprehensively evaluate the risk by bias through four domains of selection bias, information bias, confounding and multiple testing and replication, it may still not cover all possible sources of bias. We hope more researchers apply our tool in future systematic reviews of genetic studies and make suggestions to extend and refine it if needed.

Our systematic review revealed significant associations between the genes *CD14*, *FER*, and mortality/survival, as well as *FLT1* and respiratory failure among critically ill patients. However, the reliance on predominantly candidate gene studies in this field is a significant limitation. The scarcity of high-quality studies highlights the urgent need for well-designed studies with low risk of bias and well powered GWAS to advance our understanding of the genetic underpinnings of critical illness, and to move beyond the limitations of outdated candidate gene approaches. The identified associations should be prioritized for validation in such studies.

## Contributors

WZ contributed to design of the protocol, searching literature, extracting data, analysing and interpreting the data, draughting the manuscript. NNH, SR, AZ, and YY contributed to extracting data. EC contributed to design of the protocol and extracting data. These authors have accessed and verified the underlying data. ESP, CF, AYC, AM, CH shared the GWAS data of the original studies. GL, FK and HS contributed to design of the protocol and supervised, reviewed and approved the final version of the manuscript. All authors read and approved the final version of the manuscript.

## Data sharing statement

All the databases searched are publicly available. All search results and extracted data are presented in Supplementary File 2. Upon reasonable request, the code used in the analyses can be shared with other researchers by contacting the corresponding authors.

## Declaration of interests

All authors declare that they have no conflicts of interest.

## Appendix A Supplementary data

Supplementary File 1 Complete set of Supplementary Tables.

Supplementary File 2 Categorization of included phenotypes.

Supplementary File 3 Data extraction from all included studies.

Supplementary File 4 Summary of associations between outcomes, genes, and SNPs.

Supplementary File 5 Details of excluded studies.

Supplementary File 6 Significant associations between outcomes and genes.
