# Supplementary material for: Genetic association studies in critically ill patients: a systematic review
Source: eBioMedicine. 2025 Mar 31;114:105678. doi: 10.1016/j.ebiom.2025.105678 (PMC11999069; doi:10.1016/j.ebiom.2025.105678)
Supplement: Supplementary File 1 — Complete set of Supplementary Tables. [file mmc1.pdf]

Supplementary File 1. Outline

Content	Page
Table 1. PRISMA 2020 checklist	1-2
Table 2. Search strategy	3-7
Table 3. Eligibility criteria	8
Table 4. Risk of bias assessment for genetic association studies based on the *HuGE Review Handbook V1.0*	9
Table 5. Cross-checking the GWAS results	10

Table 1: PRISMA 2020 checklist

Section and Topic	Item #	Checklist item	Location where item is reported
**TITLE**			
Title	1	Identify the report as a systematic review.	Lines 1
**ABSTRACT**			
Abstract	2	See the PRISMA 2020 for Abstracts checklist.	Lines 31-55
**INTRODUCTION**			
Rationale	3	Describe the rationale for the review in the context of existing knowledge.	Lines 80-106
Objectives	4	Provide an explicit statement of the objective(s) or question(s) the review addresses.	Lines 107-112
**METHODS**			
Eligibility criteria	5	Specify the inclusion and exclusion criteria for the review and how studies were grouped for the syntheses.	Lines 128-147
Information sources	6	Specify all databases, registers, websites, organisations, reference lists and other sources searched or consulted to identify studies. Specify the date when each source was last searched or consulted.	Lines 120-126
Search strategy	7	Present the full search strategies for all databases, registers and websites, including any filters and limits used.	Supplementary File 1, Table 2
Selection process	8	Specify the methods used to decide whether a study met the inclusion criteria of the review, including how many reviewers screened each record and each report retrieved, whether they worked independently, and if applicable, details of automation tools used in the process.	Lines 150-151
Data collection process	9	Specify the methods used to collect data from reports, including how many reviewers collected data from each report, whether they worked independently, any processes for obtaining or confirming data from study investigators, and if applicable, details of automation tools used in the process.	Lines 173-190
Data items	10a	List and define all outcomes for which data were sought. Specify whether all results that were compatible with each outcome domain in each study were sought (e.g. for all measures, time points, analyses), and if not, the methods used to decide which results to collect.	Lines 134-139
	10b	List and define all other variables for which data were sought (e.g. participant and intervention characteristics, funding sources). Describe any assumptions made about any missing or unclear information.	Lines 120-126
Study risk of bias assessment	11	Specify the methods used to assess risk of bias in the included studies, including details of the tool(s) used, how many reviewers assessed each study and whether they worked independently, and if applicable, details of automation tools used in the process.	Lines 162-170
Effect measures	12	Specify for each outcome the effect measure(s) (e.g. risk ratio, mean difference) used in the synthesis or presentation of results.	Lines 176-181
Synthesis methods	13a	Describe the processes used to decide which studies were eligible for each synthesis (e.g. tabulating the study intervention characteristics and comparing against the planned groups for each synthesis (item #5)).	Not applicable
	13b	Describe any methods required to prepare the data for presentation or synthesis, such as handling of missing summary statistics, or data conversions.	Not applicable
	13c	Describe any methods used to tabulate or visually display results of individual studies and syntheses.	Not applicable
	13d	Describe any methods used to synthesize results and provide a rationale for the choice(s). If meta-analysis was performed, describe the model(s), method(s) to identify the presence and extent of statistical heterogeneity, and software package(s) used.	Not applicable
	13e	Describe any methods used to explore possible causes of heterogeneity among study results (e.g. subgroup analysis, meta-regression).	Not applicable
	13f	Describe any sensitivity analyses conducted to assess robustness of the synthesized results.	Lines 202-111
Reporting bias assessment	14	Describe any methods used to assess risk of bias due to missing results in a synthesis (arising from reporting biases).	Lines 162-170
Certainty assessment	15	Describe any methods used to assess certainty (or confidence) in the body of evidence for an outcome.	Lines 120-126

Section and Topic	Item #	Checklist item	Location where item is reported
**RESULTS**			
Study selection	16a	Describe the results of the search and selection process, from the number of records identified in the search to the number of studies included in the review, ideally using a flow diagram.	Lines 220-227
	16b	Cite studies that might appear to meet the inclusion criteria, but which were excluded, and explain why they were excluded.	Lines 224-226
Study characteristics	17	Cite each included study and present its characteristics.	Supplementary File 2
Risk of bias in studies	18	Present assessments of risk of bias for each included study.	Figure 3
Results of individual studies	19	For all outcomes, present, for each study: (a) summary statistics for each group (where appropriate) and (b) an effect estimate and its precision (e.g. confidence/credible interval), ideally using structured tables or plots.	Table 1
Results of syntheses	20a	For each synthesis, briefly summarise the characteristics and risk of bias among contributing studies.	Not applicable
	20b	Present results of all statistical syntheses conducted. If meta-analysis was done, present for each the summary estimate and its precision (e.g. confidence/credible interval) and measures of statistical heterogeneity. If comparing groups, describe the direction of the effect.	Not applicable
	20c	Present results of all investigations of possible causes of heterogeneity among study results.	Not applicable
	20d	Present results of all sensitivity analyses conducted to assess the robustness of the synthesized results.	Not applicable
Reporting biases	21	Present assessments of risk of bias due to missing results (arising from reporting biases) for each synthesis assessed.	Not applicable
Certainty of evidence	22	Present assessments of certainty (or confidence) in the body of evidence for each outcome assessed.	Figure 2
**DISCUSSION**			
Discussion	23a	Provide a general interpretation of the results in the context of other evidence.	Lines 298-347
	23b	Discuss any limitations of the evidence included in the review.	Lines 367-375
	23c	Discuss any limitations of the review processes used.	Lines 367-371
	23d	Discuss implications of the results for practice, policy, and future research.	Lines 371-375
**OTHER INFORMATION**			
Registration and protocol	24a	Provide registration information for the review, including register name and registration number, or state that the review was not registered.	Lines 35-36
	24b	Indicate where the review protocol can be accessed, or state that a protocol was not prepared.	Lines 114-118
	24c	Describe and explain any amendments to information provided at registration or in the protocol.	Lines 386-391
Support	25	Describe sources of financial or non-financial support for the review, and the role of the funders or sponsors in the review.	Lines 213-217
Competing interests	26	Declare any competing interests of review authors.	Lines 410-411
Availability of data, code and other materials	27	Report which of the following are publicly available and where they can be found: template data collection forms; data extracted from included studies; data used for all analyses; analytic code; any other materials used in the review.	Supplementary File 2-4

From: Page MJ, McKenzie JE, Bossuyt PM, Boutron I, Hoffmann TC, Mulrow CD, et al. The PRISMA 2020 statement: an updated guideline for reporting systematic reviews. BMJ 2021;372:n71. doi: 10.1136/bmj.n71

For more information, visit: http://www.prisma-statement.org/

Table 2: Search strategy

Pubmed Search Strategy

("Critical Care"[MeSH Terms] OR "Intensive Care Units"[MeSH Terms] OR icu[Title/abstract] OR “intensive care”[Title/abstract] OR icus[Title/abstract] OR “critically ill*”[Title/abstract] OR “critical ill*”[Title/abstract] OR “critical care”[Title/abstract] OR “major trauma”[Title/abstract] OR “major blunt trauma”[Title/abstract])

AND

(“Genome-Wide Association Study”[MeSH Terms] OR "Genetic Association Studies"[MeSH Terms] OR “Polymorphism, Genetic”[MeSH Terms] OR Genomics[MeSH Terms] OR "Genetic Predisposition to Disease"[MeSH Terms] OR "Genetic Research"[MeSH Terms] OR Genome[MeSH Terms] OR genetics[Mesh major topic] OR genetics[Mesh Subheading] OR “GWA stud*”[Title/abstract] OR “GWAS”[Title/abstract] OR polymorphism*[Title/abstract] OR gene[Title/abstract] OR genes[Title/abstract] OR genet*[Title/abstract] OR genom*[Title/abstract] OR genotyp*[Title/abstract] OR snp[Title/abstract] OR snps[Title/abstract] OR haplotyp*[Title/abstract] OR “genome-wide association stud*” [Title/abstract] OR “candidate gene analys*”[Title/abstract] OR “candidate gene association stud*”[Title/abstract] OR “whole genome sequenc*”[Title/abstract] OR “whole exome sequenc*”[Title/abstract])

AND

("Multiple Organ Failure"[MeSH Terms] OR "Shock, Septic"[MeSH Terms] OR Sepsis[Mesh Terms] OR "Liver Failure"[MeSH Terms] OR "Heart Failure"[MeSH Terms] OR "Respiratory Distress Syndrome"[MeSH Terms] OR "Pulmonary Edema"[MeSH Terms] OR "Acute Lung Injury"[MeSH Terms] OR "Renal Insufficiency"[Mesh major topic] OR “multiple organ failure*”[Title/abstract] OR MODS[Title/abstract] OR “multiple organ dysfunction*”[Title/abstract] OR “multi-organ*”[Title/abstract] OR “organ failure*”[Title/abstract] OR “organ dysfunction*”[Title/abstract] OR “liver failure*”[Title/abstract] OR “heart failure*”[Title/abstract] OR “cardiac dysfunction*”[Title/abstract] OR “left ventricular dysfunction*”[Title/abstract] OR sepsis[Title/abstract] OR “septic shock”[Title/abstract] OR “renal insufficienc*”[Title/abstract] OR “acute kidney injur*”[Title/abstract] OR AKI[Title/abstract] OR “acute lung injur*”[Title/abstract] OR “pulmonary edema”[Title/abstract] OR “acute respiratory distress syndrome”[Title/abstract] OR ARDS[Title/abstract] OR “respiratory failure*”[Title/abstract] OR "Coronavirus Infections"[MeSH Terms] OR covid*[Title/Abstract] OR corona[Title/Abstract] OR coronavirus[Title/abstract] OR sars-CoV-2[Title/Abstract])

AND

("Cohort Studies"[MeSH Terms] OR "Case-Control Studies"[MeSH Terms] OR "cohort*"[Title/Abstract] OR "case-control"[Title/Abstract] OR "case control"[Title/Abstract])

NOT

("Animals"[MeSH Terms] NOT "Humans"[MeSH Terms]) NOT ("Review" [Publication type] OR "Case reports" [Publication type] OR "Meta-analysis" [Publication type] OR review[Title] OR "case report" [Title] OR "meta-analysis" [Title] OR "meta analys*" [Title]) NOT (("Child"[MeSH Terms] OR "Adolescent"[MeSH Terms] OR "Infant"[MeSH Terms] OR neonat*[Title] OR pediatric*[Title]) NOT "Adult"[MeSH Terms])

Embase Search Strategy

('intensive care'/exp OR 'critical care'/exp OR 'intensive care unit'/exp OR 'intensive care':ab,ti,kw OR ICU:ab,ti,kw OR ICUs:ab,ti,kw OR 'critically ill*':ab,ti,kw OR 'critical ill*':ab,ti,kw OR 'critical care':ab,ti,kw OR 'major trauma':ab,ti,kw OR 'major blunt trauma':ab,ti,kw)

AND

('genome-wide association study'/exp OR 'genetic association study'/exp OR genomics/exp OR genome/exp OR 'genetic predisposition'/exp OR 'genetic polymorphism'/exp OR genetics/mj OR 'candidate gene'/exp OR 'whole genome sequencing'/exp OR 'whole exome sequencing'/exp OR polymorphism*:ab,ti,kw OR gene:ab,ti,kw OR genes:ab,ti,kw OR genet*:ab,ti,kw OR genom*:ab,ti,kw OR genotyp*:ab,ti,kw OR snp:ab,ti,kw OR snps:ab,ti,kw OR haplotyp*:ab,ti,kw OR 'genome-wide association stud*':ab,ti,kw OR 'GWA stud*':ab,ti,kw OR 'GWAS':ab,ti,kw OR 'candidate gene analys*':ab,ti,kw OR 'candidate gene association stud*':ab,ti,kw OR 'whole genome sequenc*':ab,ti,kw OR 'whole exome sequenc*':ab,ti,kw)

AND

('multiple organ failure'/exp OR sepsis/exp OR 'septic shock'/exp OR 'liver failure'/exp OR 'kidney failure'/exp OR 'heart failure'/exp OR 'respiratory distress syndrome'/exp OR 'lung edema'/exp OR 'acute lung injury'/exp OR 'multiple organ failure*':ab,ti,kw OR MODS:ab,ti,kw OR 'multiple organ dysfunction*':ab,ti,kw OR 'multi-organ*':ab,ti,kw OR 'organ failure*':ab,ti,kw OR 'organ dysfunction*':ab,ti,kw OR 'liver failure*':ab,ti,kw OR 'heart failure*':ab,ti,kw OR 'cardiac dysfunction*':ab,ti,kw OR 'left ventricular dysfunction*':ab,ti,kw OR sepsis:ab,ti,kw OR 'septic shock':ab,ti,kw OR 'renal insufficienc*':ab,ti,kw OR 'acute kidney injur*':ab,ti,kw OR AKI:ab,ti,kw OR 'acute lung injur*':ab,ti,kw OR 'pulmonary edema':ab,ti,kw OR 'acute respiratory distress syndrome':ab,ti,kw OR ARDS:ab,ti,kw OR 'respiratory failure*':ab,ti,kw OR 'Coronavirus Infection'/exp OR covid*:ab,ti,kw OR corona:ab,ti,kw OR coronavirus:ab,ti,kw OR sars-CoV-2:ab,ti,kw)

AND

('cohort analysis'/exp OR 'case control study'/exp OR 'cohort*':ab,ti,kw OR 'case-control':ab,ti,kw OR 'case control':ab,ti,kw)

NOT

(animal/exp NOT human/exp) NOT (Review:it OR 'Case reports':it OR 'Case report':it OR 'Meta-analysis':it OR meta-analysis:it OR 'conference abstract':it OR 'review':ti

OR ‘case report’:ti OR ‘meta-analysis’:ti OR ‘meta analys*’:ti) NOT (('child'/exp OR 'adolescent'/exp OR 'infant'/exp OR neonat*:ti OR pediatric*:ti) NOT 'adult'/exp)

Cochrane Library Search Strategy

#1 MeSH descriptor: [Critical Care] explode all trees

#2 MeSH descriptor: [Intensive Care Units] explode all trees

#3 ((intensive care unit):ti,ab,kw OR (ICU):ti,ab,kw OR (ICUs):ti,ab,kw OR (critically ill*):ti,ab,kw OR (critical ill*):ti,ab,kw OR (critical care):ti,ab,kw OR (major trauma):ti,ab,kw OR (major blunt trauma):ti,ab,kw)

#4 MeSH descriptor: [Genome-Wide Association Study] explode all trees

#5 MeSH descriptor: [Genetic Association Studies] explode all trees

#6 MeSH descriptor: [Genome] in all MeSH products

#7 MeSH descriptor: [Genetic Predisposition to Disease] explode all trees

#8 MeSH descriptor: [Polymorphism, Genetic] explode all trees

#9 MeSH descriptor: [Genetic Association Studies] explode all trees

#10 MeSH descriptor: [Whole Genome Sequencing] explode all trees

#11 MeSH descriptor: [Exome Sequencing] explode all trees

#12 ((polymorphism*):ti,ab,kw OR (gene):ti,ab,kw OR (genes):ti,ab,kw OR (genet*):ti,ab,kw OR (genom*):ti,ab,kw OR (genotyp*):ti,ab,kw OR (SNP):ti,ab,kw OR (SNPs):ti,ab,kw OR (haplotyp*):ti,ab,kw OR (genome-wide association study):ti,ab,kw OR (candidate gene analys*):ti,ab,kw OR (candidate gene association stud*):ti,ab,kw OR (whole genome sequenc*):ti,ab,kw OR (whole exome sequenc*):ti,ab,kw)

#13 MeSH descriptor: [Multiple Organ Failure] explode all trees

#14 MeSH descriptor: [Sepsis] explode all trees

#15 MeSH descriptor: [Shock, Septic] explode all trees

#16 MeSH descriptor: [Liver Failure] explode all trees

#17 MeSH descriptor: [Renal Insufficiency] explode all trees

#18 MeSH descriptor: [Heart Failure] explode all trees

#19 MeSH descriptor: [Respiratory Distress Syndrome] explode all trees

#20 MeSH descriptor: [Acute Lung Injury] explode all trees

#21 ((multiple organ failure*):ti,ab,kw OR (MODS):ti,ab,kw OR (multiple organ dysfunction*):ti,ab,kw OR (multi organ*):ti,ab,kw OR (organ failure*):ti,ab,kw OR (organ dysfunction*):ti,ab,kw OR (liver failure*):ti,ab,kw OR (heart failure*):ti,ab,kw OR (cardiac dysfunction*):ti,ab,kw OR (left ventricular dysfunction*):ti,ab,kw OR (sepsis):ti,ab,kw OR (septic shock):ti,ab,kw OR (renal insufficienc*):ti,ab,kw OR (acute kidney injur*):ti,ab,kw OR (AKI):ti,ab,kw OR (acute lung injur*):ti,ab,kw OR (pulmonary edema):ti,ab,kw OR (acute respiratory distress syndrome):ti,ab,kw OR (ARDS):ti,ab,kw OR (respiratory failure*):ti,ab,kw OR (covid*):ti,ab,kw OR (corona):ti,ab,kw OR (coronavirus):ti,ab,kw OR (sars-CoV-2):ti,ab,kw)

#22 MeSH descriptor: [Cohort Studies] explode all trees

#23 MeSH descriptor: [Case-Control Studies] explode all trees

#24 ((cohort*):ti,ab,kw OR (case-control):ti,ab,kw OR (“case control”):ti,ab,kw)

#25 MeSH descriptor: [Animal Experimentation] explode all trees

#26 MeSH descriptor: [Human Experimentation] explode all trees

#27 ((“review”):pt OR (“case report”):pt OR (“meta analysis”):pt OR (“abstract”):pt OR (review):ti OR (case report):ti OR (meta-analysis):ti OR (meta analys*):ti)

#28 (((child):kw OR (infant):kw OR (infant*):ti OR (neonat*):ti OR (pediatric*):ti) NOT (adult):kw)

(#1 OR #2 OR #3) AND (#4 OR #5 OR #6 OR #7 OR #8 OR #9 OR #10 OR #11 OR #12) AND (#13 OR #14 OR #15 OR #16 OR #17 OR #18 OR #19 OR #20 OR #21) AND (#22 OR #23 OR #24) NOT (#25 NOT #26) NOT #27 NOT #28

Table 3: Eligibility criteria

Inclusion	Exclusion
1. All types of genetic association studies (e.g. candidate gene studies, genome-wide association studies)	1. Studies without original data or enough information (e.g. review, meta-analysis, conference abstracts, case reports/series, editorials)
2. Studies including adult (≥ 18 years old) critically ill patients	2. Association studies of (genome-wide) methylation or gene expression
3. Studies including patients who developed mortality multiple organ failure, heart failure, liver failure, acute kidney injury, renal insufficiency, sepsis, septic shock, acute respiratory distress syndrome, pulmonary oedema, acute lung injury, and COVID-19	3. Studies including patients with other diseases than mentioned previously (e.g. neurological diseases such as stroke or subarachnoid hemorrhage)
4. Studies reporting any other patient-relevant outcome measure was reported for the primary analysis	4. Studies including patients admitted to the ICU after planned surgery
	5. Studies only reporting associations with surrogate outcomes or laboratory values (e.g., leucocytes, proteins, or cytokines)

Table 4: Risk of bias assessment for genetic association studies based on the *HuGE Review Handbook V1.0*

	Y	PY	NI	PN	N
**1.Domain Selection bias**					
Biased selection of cases<br>1.1: Did the study describe the selection process of the cases?					
Biased selection of controls<br>1.2: Are the controls selected from the same source population as the case-subjects?					
Differential participation in cases and controls<br>1.3: Was the proportion of non-participation similar in cases and controls?					
Loss to follow-up<br>1.4: The study did not suffer from substantial loss to follow up and/or the proportion of loss of follow-up similar in cases and controls.					
**2. Domain Information bias**					
2.1: Was genotyping of cases and controls performed together (not separately)?					
2.2: *For candidate genes studies:* Were the laboratory staff and outcome assessors blinded to the genotyping results and outcome of interest?					
2.3 *For genome-wide association studies:* Was the genotyping array appropriate for the ethnic group?					
Quality control of genotypes<br>2.4: *For candidate gene studies*: Was the departure from Hardy-Weinberg Equilibrium (HWE) tested (in controls) and its significance/magnitude of departure evaluated?					
2.5: *For genome-wide association studies*: Did the study apply genotype quality control measures to samples and markers (minor allele frequency, call rate, imputation quality, HWE, etc.)?					
**3. Domain Confounding**					
Population stratification<br>3.1: Did the study control for population stratification?					
"Confounding" from other sources<br>3.2: Did the study adjust for age and/or sex?					
**4. Domain Multiple testing and replication**					
4.1: Did the study adjust for multiple testing?					
4.2: Did the study try to replicate the results?					

Abbreviations: Y: yes; PY: probably yes; NI: no information; PN: probably no; N: no

**Each domain has signaling questions. Only if answers to all signaling questions within a domain are 'yes' or 'probably yes', then the domain will be assessed as 'low risk of bias'. If the answers to any of the signaling questions within a domain are 'no information', then the domain will be assessed as 'some concerns'. If one or more of the answers to signaling questions within a domain are 'probably no' or 'no', then the domain will be assessed as 'high risk of bias'.*

We will consider studies that are assessed as having 'low risk of bias' in all domains as being at 'overall low risk of bias'. Studies with one or more of these domains assessed as 'some concerns' or 'high risk of bias' will be judged as being at 'overall high risk of bias'.

Table 5: Cross-checking the GWAS results

SNP	Original study	Trait in the oringinal study	Cross-checking study	Trait in the cross-checking study	Chromosome	Position	Effect allele	Non-effect allele	Effect allele frequency	Beta	Se	p
rs4957796	Rautanen A, Lancet Respir Med, 2015	28-day mortality/survival in sepsis patients	Guillen-Guio B, Lancet Respir Med, 2020	ARDS	5	108402140	C	T	0.16	-0.03	0.16	0.84
rs79423885	Rautanen A, Lancet Respir Med, 2015	28-day mortality/survival in sepsis patients	Guillen-Guio B, Lancet Respir Med, 2020	ARDS	6	103810003	G	A	0.09	-0.19	0.21	0.36
rs2734600	Guillen-Guio B, Lancet Respir Med, 2020	ARDS	Rautanen A, Lancet Respir Med, 2015	28-day mortality/survival in sepsis patients	9	33753355	C	T	0.13	0.01	0.16	0.93
rs11195238	Guillen-Guio B, Lancet Respir Med, 2020	ARDS	Rautanen A, Lancet Respir Med, 2015	28-day mortality/survival in sepsis patients	10	112388857	C	T	0.12	-0.11	0.18	0.54
rs9508032	Guillen-Guio B, Lancet Respir Med, 2020	ARDS	Rautanen A, Lancet Respir Med, 2015	28-day mortality/survival in sepsis patients	13	28995940	C	T	0.26	-0.02	0.13	0.87
rs8001184	Guillen-Guio B, Lancet Respir Med, 2020	ARDS	Rautanen A, Lancet Respir Med, 2015	28-day mortality/survival in sepsis patients	13	90603540	C	A	0.50	0.03	0.11	0.80
