# Supplementary figures and images for: Genetic association studies in critically ill patients: a systematic review
Source: eBioMedicine. 2025 Mar 31;114:105678. doi: 10.1016/j.ebiom.2025.105678 (PMC11999069; doi:10.1016/j.ebiom.2025.105678)

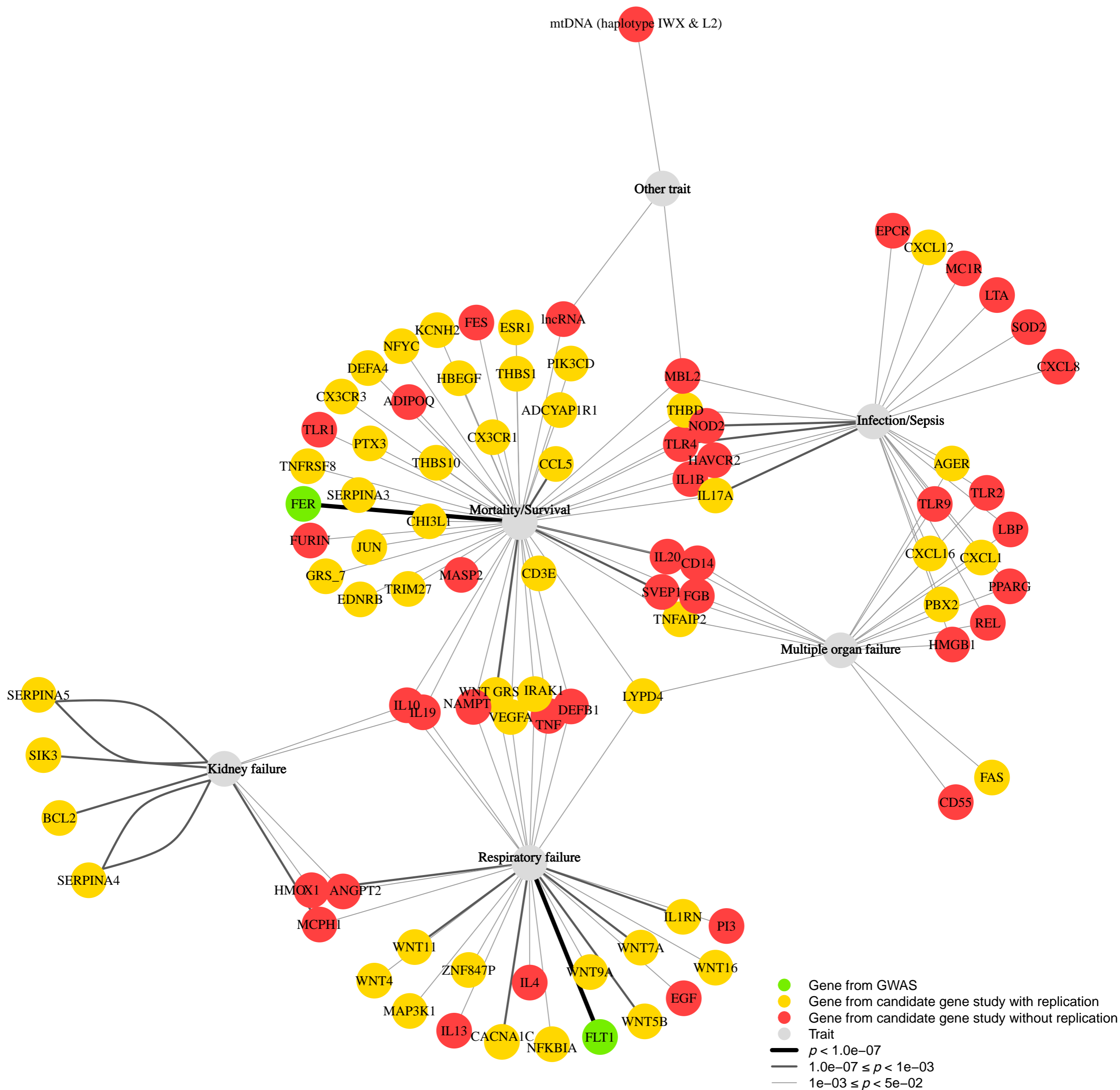

Supplement: Supplementary File 6 — Significant associations between outcomes and genes. [file mmc6.pdf]
